# Prognostic factors and treatment outcomes in patients with Small Bowel Adenocarcinoma (SBA): The Royal Marsden Hospital (RMH) experience

**DOI:** 10.1186/s12885-015-1014-6

**Published:** 2015-01-21

**Authors:** Khurum Khan, Clare Peckitt, Francesco Sclafani, David Watkins, Sheela Rao, Naureen Starling, Vikram Jain, Sachin Trivedi, Susannah Stanway, David Cunningham, Ian Chau

**Affiliations:** Department of Medicine, GI and Lymphoma Unit, The Royal Marsden NHS Foundation Trust, London and Surrey, UK

## Abstract

**Background:**

SBA is a rare tumour which carries a poor prognosis. Very few data on prognostic factors and treatment outcomes are available. We conducted a retrospective analysis of patients treated for SBA at our institution.

**Methods:**

Clinico-pathological characteristics, treatments and outcomes of all the SBA patients treated consecutively from 1996 to 2011 were retrospectively collected. The prognostic value of baseline factors was assessed using the Cox regression model. The Kaplan-Meier method was used to estimate the survival outcomes.

**Results:**

Eighty-four patients with SBA were treated during the study period. Of these, 48 presented with early stage SBA, while 36 had unresectable disease. All early stage SBA patients (58.3% males; median age, 59 years) underwent resection (R0 in 44/48) and 27 (56%) received adjuvant chemotherapy. Median relapse-free survival and overall survival (OS) were 31.1 months (95% CI: 8.0-54.3) and 42.9 (95% CI: 0–94.9), respectively. In univariate analyses, poor histological differentiation (p = 0.025) and lymphovascular invasion (p = 0.003) were prognostic for OS. In the group of patients with relapsed, unresectable or metastatic disease (n = 59), systemic chemotherapy was administered in 46 cases (78%). The response rate to first line chemotherapy was 50%. Median progression-free survival and OS were 8.8 (95% CI: 5.5-12.3) and 12.8 months (95% CI: 8.4-17.2), respectively. In univariate analyses, low albumin (p = 0.041) and high platelet count (p = 0.007) were prognostic for OS.

**Conclusion:**

Prospective clinical trials are needed to inform the management of SBA patients. Prognostic factors evaluated in our series may be useful for patient stratification and treatment selection in future studies.

## Background

Although small bowel accounts for 75% of the total length of the entire intestine, tumours arising from the small intestine are relatively rare [1], accounting for ≤5% of all gastrointestinal tract cancers. Small bowel adenocarcinoma (SBA) is the second commonest tumour involving the small bowel after carcinoid tumours [1]. The estimated new cases per year in US and Europe are 5300 and 3500 respectively with estimated mortality of 1,210 and 1100 deaths per year [2,3].

The outcome from SBA remains poor across all stages, with a median overall survival (OS) of about 19 months and an overall 5-year OS of around 30% [4]. More importantly, given the rarity of this disease, there is a lack of data from randomised, prospective studies and the management of these tumours is generally based on data from small retrospective series or is inferred from the available evidence on other tumour types.

Indeed, due to its anatomical proximity to large bowel and the presence of some clinico-pathological commonalities with colorectal cancer (CRC), SBA is often assimilated to the adenocarcinoma arising from the large bowel and largely treated according to the management recommendations for CRC. However, the natural history of the two diseases is significantly different. It is recognised that SBA and CRC differ for their clinical features and tumour-related symptoms at presentation. Moreover, it is thought that the impact of surgery and chemotherapy (both in the adjuvant and metastatic setting) [5,6] in SBA is significantly lower than in CRC, perhaps suggesting that SBAs may have a more aggressive phenotype than CRCs and are less sensitive to the treatments used for CRC. Finally, as a result of the low prevalence of this disease and the scarcity of clinical data, there are no validated prognostic factors or tumour biomarkers for SBA which may help clinicians stratify patients according to their individual risk profile, predict the clinical benefit of specific treatment strategies and accurately monitor the course of disease.

In this scenario, prospective clinical trials which provide high level evidence to inform the management of SBA both in the early stage and metastatic setting are urgently needed. However, until the results of these studies are available, clinical data from retrospective series may offer useful insights into the natural history and treatment outcomes of SBA.

In this article, we report the clinical characteristics and outcomes of patients treated consecutively for SBA at The Royal Marsden Hospital over a fifteen year period.

## Methods

This retrospective study included all patients with SBA consecutively treated at the Royal Marsden National Health Service (NHS) Foundation Trust, United Kingdom, from January 1996 to December 2011. Only patients who had confirmed histological diagnosis of SBA were included. Patient medical records were reviewed and the following clinico-pathologic parameters were collected for all patients included in the study: age, gender, site of origin of the primary tumour, histological subtype, tumour grade, clinical stage at diagnosis, presenting features including weight loss (defined as unintentional loss of body weight of at least 10%, during the last six months), full blood count, biochemical profiles (including alkaline phosphatase [ALP], bilirubin, lactate dehydrogenase [LDH] and albumin) and CEA. For patients undergoing resection of the primary tumour, type of surgery (R0 vs. R1 vs. R2), lymphovascular invasion (LVI), perineural invasion (PNI), number of lymph nodes harvested (LNH), and number of lymph nodes involved (LNI) were also collected. Details on systemic treatments were collected for the whole study population. Patients were divided into two groups; those with early stage SBA (ES-SBA) included patients who presented with potentially resectable disease and late stage SBA (LS-SBA). LS-SBA included patients who presented or relapsed with un-resectable disease at a later stage.

The study was approved by the local Research Ethics Committee.

### Clinical presentation and response evaluation

Clinical symptoms at initial presentation were collected as originally recorded and prospectively reported in the electronic medical records (EMR). Baseline tumour measurements were performed within 2–4 weeks prior to treatment start in all the patients included in this study. In patients with ES-SBA, as per policy of our institution, surveillance guidelines included annual tumour assessments by computed tomography (CT) scan for first three years, followed by clinical monitoring for subsequent two years. In patients with LS-SBA, tumour measurements were repeated every 12 weeks or earlier if progression of disease (PD) was suspected. All the scans were reported using, Response Evaluation Criteria In Solid Tumours (RECIST) version 1.0. Tumour responses were confirmed prospectively by a radiologist. Whole body PET scans were performed only when clinically indicated. Survival data were obtained from the hospital EMR, and when necessary, by contacting the general practitioner or referring institution.

### Statistical methods

The study endpoints were relapse free survival (RFS) and overall survival (OS), in ES-SBA; while in LS-SBA, progression free survival (PFS) and OS were the study endpoints. RFS was defined as the time between the date of surgery to date of relapse or death in patients who underwent R0 resection; PFS was defined as the time from date of diagnosis to date of progression or death and, OS as the date of diagnosis to date of death. Patients event-free were censored at date of last follow-up. In LS-SBA, response rate (RR) to chemotherapy was also established. Complete response (CR) was defined as complete disappearance of the tumor. Partial response (PR) was defined as more than 30% decrease in the maximum diameter of measurable disease, in the absence of progression in non-target lesions or new disease. Progressive disease (PD) was defined as more than a 20% increase in the maximum diameter of measurable disease, or as evidence of new disease or progression in non-target lesions. Stable disease (SD) was defined as the disease that did not fit the category of PR or PD [7]. Treatment was stopped in patients with radiological evidence of disease progression according to the above-mentioned criteria. Association to baseline prognostic factors were sought by performing Cox regression univariate analysis (UVA).

Categorisation of numeric laboratory variables was undertaken based on considerations of the standard reference values (normal range versus low/elevated) or according to the median values. Survival estimates and 95% confidence intervals [CI] were determined using the Kaplan-Meier method, and estimates between groups were compared using the log-rank test for baseline prognostic factors. Cox regression analysis was used to calculate respective hazard ratios and 95% CIs. Multivariate Cox regression was used to test independence of significant (p < 0.1) factors in univariate analysis. P-values <0.05 were considered significant.

## Results

### Patient and tumour characteristics

A total of 84 patients with SBA were treated at the RMH, during the study time period. Forty eight and 59 patients had ES-SBA and LS-SBA respectively; 23 patients from ES-SBA relapsed later and became un-resectable and thus were also included in LS-SBA group. The median age at presentation in ES-SBA and LS-SBA were 59 (mean = 57, range 28–78) and 61 (mean = 59, range 28–87) respectively. Most patients had duodenal tumours in both groups; 62.5% in ES-SBA and 67.8% in LS-SBA. In ES-SBA, 44 (91.6%) patients underwent R0 resection of their disease, while 27 (55.1%) patients received adjuvant chemotherapy. Of the patients with LS-SBA, 54 (91.5%) had metastatic and 5(8.5%) had localised but un-resectable disease. Forty six (78%) patients received first line chemotherapy. A summary of all the baseline features has been provided in Table 1.

**Table 1:** **Demographics/tumour characteristics**

	ES-SBA	LS-SBA
	N (%) = 48	N (%) = 59
Gender		
Male	28 (58.3)	31 (52.5)
Female	20 (41.7)	28 (47.5)
Age (mean & range)	57 (28–78)	59 (28 – 87)
Median	59	61
Site of Primary tumour		
Duodenum	30 (62.5)	40 (67.8)
Jejunum	10 (20.8)	11 (18.6)
Ileum	7 (14.6)	8 (13.6)
Small bowel unknown	1 (2.1)	0 (0)
Grade (differentiation)		
Low and moderate	27 (56.3)	N/A
Poor	20 (41.7)	
Missing	1 (2.1)	
Nodes harvested		
≤12	21 (43.8)	N/A
>12	16 (33.3)	
Missing	11 (22.9)	
Nodes involved (in those harvested)		N/A
Negative (stage I/II)	30 (62.5)	
Positive (Stage III)	12 (25.0)	
Missing	6 (12.5)	
LVI		
No	18 (37.5)	N/A
Yes	22 (45.8)	
Missing	8 (16.7)	
PNI		
No	29 (60.4)	N/A
Yes	11 (22.9)	
Missing	8 (16.7)	
Resection Status		
R0	44 (91.6)	N/A
R1	4 (8.4)	
Adjuvant Chemo		
No	21 (44.9)	N/A
Yes	27 (55.1)	
Single	9 (18.8)	
Multiple	18 (37.5)	
Disease status		
Locally Advanced	N/A	5 (8.5)
Metastatic		54 (91.5)
First Line Chemo		
No	N/A	13 (22.0)
Yes		46 (78.0)

### Presenting features

Abdominal pain was the commonest presenting symptom in both ES and LS-SBA, accounting for 34.3% and 55.3% of the initial presenting symptoms of the two groups respectively. Other common presenting symptoms in the two groups included nausea, vomiting, small bowel obstruction and weight loss. A small proportion of patients presented with haematemesis or melaena (Table 2). In ES-SBA, 44.7% of the patients presented with abnormal CEA, low haemoglobin (63.2%), low albumin (42.1%), high LDH (48%), high ALP (36.8%) and abnormal ALT (21.6%). In LS-SBA, the abnormal laboratory parameters included, low haemoglobin (78.4%), high CEA (75.6%), low albumin (47.1%), high LDH (43.9%), high ALP (43.1%) and high ALT (20%).

**Table 2:** **Symptoms present at diagnosis**

	ES-SBA	LS-SBA
	%	%
Abdominal Pain	34.3	55.3
Nausea	27.8	41.3
Vomiting	27.8	38.3
Small Bowel Obstruction	22.2	19.1
Weight loss	19.4	38.3
Jaundice	8.3	14.9
Haematemesis	8.3	8.5
Melaena	2.8	4.3
Acute abdomen perforation	0	0

### Survival outcomes

The median RFS in patients with ES-SBA after a median follow up of 76.4 (95% CI = 39.3 – 113.5) months, defined by the interval between the date of surgery to relapse or death in patients with R0 resection was 29.6 months (95% CI = 3.3-55.9). The OS defined by date of diagnosis to date of death was 42.9 months (95% CI = 0-94.9). The 5-year RFS and OS were 34.1% (95% CI = 19.0-49.2) and 47.7% (95% CI = 31.2-64.2) respectively.

**Figure 1:**
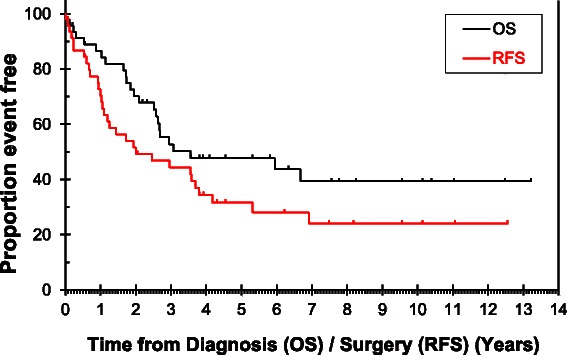
**Overall survival and relapse free survival in early stage-SBA.**

**Figure 2:**
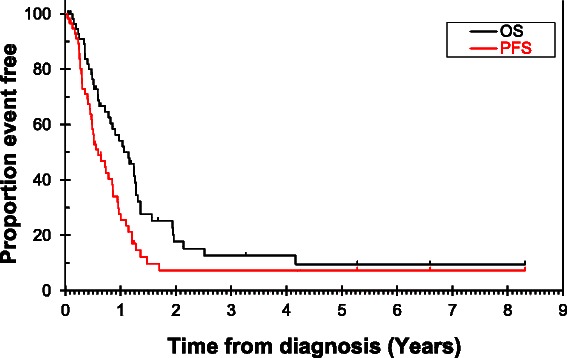
**Overall survival and progression free survival in late stage-SBA.**

In the LS-SBA group the median PFS was found to be 8.8 months (95% CI = 5.5-12.3) and OS was 12.8 months (CI = 8.4-17.2), after a median duration of follow up of 63.7 months (95% CI = 16.5-110.9) (Figures 1 and 2).

### Prognosticators of survival

Poor histological differentiation, abnormal CEA at presentation and LVI were prognostic of OS and for RFS in ES-SBA, using univariate cox regression analysis. These results are summarised in Tables 3 and 4. In multivariate analysis including any factors with p < 0.1 only LVI remained significant for OS at p < 0.05 and primary tumour (p = 0.02) and grade (0.006) remained significant for RFS, however this is based on a model including only 35 subjects due to missing data.

**Table 3:** **ES-SBA - relapse free survival by prognostic factors**

		Number events/Number subjects	5 Year survival (95% CI)	Hazard ratio (95% CI)	p-value
Gender	Male	16/24	23.1 (3.7 – 42.5)	1.58 (0.74 – 3.36)	0.236
	Female	12/20	45.0 (23.2 – 66.8)	-	
Primary tumour		13/27	54.3 (34.3 – 74.3)	-	(p = 0.019)
	Duodenum	8/9	11.1 (0 – 31.7)	1.91 (0.78 – 4.65)	0.154
	Jejunum	6/7	0	3.54 (1.28 – 9.7)	0.014
	Ileum	1/1	0		0.016
Small bowel unknown				15.9 (1.69 – 150)	
Grade (differentiation)	Low/Moderate	13/27	47.2 (26.4 – 68.0)	-	
	Poor	14/16	15.0 (0 – 33.6)	3.16 (1.43 – 6.97)	0.004
Nodes harvested	<12	15/20	50.3 (30.3 – 70.3)	-	
	≥12	4/14	74.1 (48.8 – 99.4)	0.41 (0.14 – 1.24)	0.113
Nodes involved	Negative (Stage I/II)	15/30	40.8 (21.2 – 60.4)	-	
		8/9		1.90 (0.80 - 4.51)	0.144
	Positive (Stage III)		33.3 (2.5 – 64.1)		
LVI	No	7/18	55.1 (27.9 – 82.3)	-	
	Yes	15/19	25.3 (5.3 – 45.3)	3.18 (1.28 – 7.89)	0.013
PNI	No	14/27	38.7 (18.3 – 59.1)	-	
	Yes	8/10	40.0 (9.6 – 70.4)	1.27 (0.53 – 3.06)	0.589
Adjuvant Chemo	No	11/20	42.3 (18.8 – 65.8)	-	
	Yes	17/24	28.1 (8.9 – 47.3)	1.26 (0.58 – 2.74)	0.555
Small Bowel Obstruction	None	14/25	42.8 (22.2 – 63.4)	-	
	Present	6/8	0	1.95 (0.74 – 5.15)	0.180
Weight loss	None	16/27	36.1 (16.3 – 55.9)	-	
	Present	4/6	33.3 (0 – 70.9)	1.45 (0.48 – 4.36)	0.505
CEA at baseline	Normal	8/19	53.8 (29.5 – 78.1)	-	
	Abnormal	13/15	26.7 (4.4 – 49.0)	2.25 (0.93 – 5.46)	0.074
Haemoglobin	Normal	9/14	36.4 (9.0 – 63.8)	-	
	Abnormal	14/20	35.0 (14.0 – 56.0)	1.13 (0.49 – 2.61)	0.781
Albumin	Normal	12/19	38.5 (15.6 – 61.4)	-	
	Abnormal	11/15	33.3 (9.4 – 57.2)	1.09 (0.48 – 2.48)	0.838
LDH	Normal	8/12	33.3 (3.7 – 62.9)	-	
	Abnormal	7/11	36.4 (8.0 – 64.8)	0.88 (0.32 – 2.45)	0.808
Alk.Ph	Normal	13/21	39.8 (18.0 – 61.6)	-	
	Abnormal	10/13	30.8 (5.7 – 55.9)	1.08 (0.47 – 2.47)	0.858
ALT	Normal	16/26	40.0 (20.6 – 59.4)	-	
	Abnormal	6/7	28.6 (0 – 62.1)	1.23 (0.48 – 3.18)	0.671
Platelets	Normal	24/40	38.0 (21.7 – 54.3)	-	
	Abnormal	1/1	0	4.15 (0.52 – 33.2)	0.180

Where the normal values are:

CEA: ≤ 3.

Platelets: 150 – 400 × 10^9^/l.

Hemoglobin: Male: 13.0 – 17.0 mg/l .

Female: 12.0 - 15.0 mg/l.

Albumin: >35 g/l.

LDH: 98–192 U/l.

ALP: 24–110 U/l.

ALT: <40 U/l.

**Table 4:** **ES - SBA - overall survival BY prognostic factors**

		Number events/Number subjects	5 Year survival (95% CI)	Hazard ratio (95% CI)	p-value
Gender	Male	15/28	41.0 (19.8 – 62.2)	1.81 (0.79 – 4.15)	0.160
	Female	9/20	55.5 (33.2 – 76.8)	-	
Primary tumour	Duodenum	12/30	60.3 (40.9 – 79.7)	-	(0.046)
	Jejunum	7/10	30.0 (1.6 – 58.4)	1.61 (0.63 – 4.11)	0.316
	Ileum	4/7	33.3 (0 – 70.9)	1.97 (0.63 – 6.22)	0.246
	Small bowel unknown	1/1	0	28.4 (2.48 – 326.5)	0.007
Grade	Low/(differentiation)	10/27	56.9 (36.7 – 77.1)	-	
Moderate	Poor	14/20	34.8 (12.7 – 56.9)	2.58 (1.13 – 5.89)	0.025
Nodes harvested	<12	12/21	42.9 (21.7 – 64.1)	-	
	≥12	6/16	69.6 (44.3 – 94.9)	0.75 (0.28 – 2.01)	0.572
Nodes involved	Negative (Stage I/II)	13/30	49.7 (30.1 – 69.3)	-	
	Positive (Stage III)	9/12	41.7 (13.9 – 69.5)	1.64 (0.70 – 3.84)	0.259
LVI	No	3/18	78.6 (57.0 – 100)	-	
	Yes	17/22	30.0 (10.4 – 49.6)	6.73 (1.95 – 23.2)	0.003
PNI	No	13/29	51.4 (31.4 – 71.4)	-	
	Yes	7/11	45.5 (16.1 – 74.9)	1.42 (0.56 – 3.55)	0.460
Adjuvant Chemo	No	10/21	42.0 (18.5 – 65.5)	-	
	Yes	14/27	52.1 (32.3 – 71.9)	0.93 (0.41 – 2.11)	0.867
Small Bowel Obstruction	None	13/28	46.9 (26.9 – 66.9)	-	
	Present	5/8	29.2 (0 – 63.1)	1.75 (0.62 – 4.97)	0.294
Weight loss	None	14/29	45.0 (25.6 – 64.4)	-	
	Present	4/7	33.3 (0 – 70.9)	1.92 (0.63 – 5.84)	0.252
CEA at baseline	Normal	7/21	65.0 (44.0 – 86.0)	2.29 (0.90 – 5.82)	0.082
	Abnormal	12/17	37.5 (13.8 – 61.2)	-	
Hemoglobin	Normal	7/14	44.5 (16.5 – 72.5)	-	
	Abnormal	15/24	43.5 (23.3 – 63.7)	1.27 (0.52 – 3.31)	0.604
Albumin	Normal	12/22	46.7 (24.9 – 68.5)	-	
	Abnormal	10/16	40.0 (15.3 – 64.7)	1.14 (0.49 – 2.65)	0.756
LDH	Normal	7/13	48.0 (18.6 – 77.4)	-	
	Abnormal	7/12	50.0 (21.8 – 78.2)	0.87 (0.30 – 2.52)	0.874
Alk.Ph	Normal	14/24	43.1 (22.5 – 63.7)	-	
	Abnormal	8/14	46.2 (19.2 – 73.2)	0.75 (0.31 – 1.79)	0.512
ALT	Normal	16/29	46.0 (27.4 – 64.6)	-	
	Abnormal	5/8	42.9 (6.2 – 79.6)	1.05 (0.38 – 2.89)	0.921
Platelets	Normal	21/43	50.9 (35.0 – 66.8)	-	
	Abnormal	1/2	0	6.68 (0.77 – 57.6)	0.084

**Figure 3:**
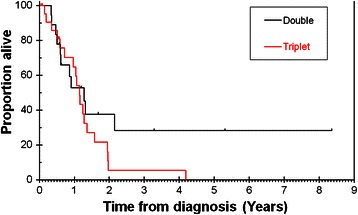
**Overall survival by doublet vs. triplet chemotherapy in late stage-SBA.**

Abnormal albumin at diagnosis (p = 0.041), platelet count (p = 0.007) and CEA levels (p = 0.025) were prognostic of OS in patients with LS-SBA, who received chemotherapy. Doublet (18/41) vs. triplet (23/41) chemotherapy regimens had no prognostic impact on OS (p = 0.185) (Figure 3). 5/41 patients received single agent chemotherapy. These results are summarised in Tables 5 and 6. In multivariate analysis including any factors with p < 0.1 only platelets remained significant for OS at p < 0.05 and only albumin remained significant for PFS, however this is based on a model including only 33 subjects due to missing data.

**Table 5:** **LS-SBA - progression free survival by prognostic factors in only patients who received first-line chemotherapy**

		Number events/Number subjects	1 Year survival(95% CI)	Hazard ratio(95% CI)	p-value
Gender	Male	20/24	20.2 (2.8 – 37.6)	1.69 (0.88 – 3.24)	0.115
	Female	17/22	39.4 (17.3 – 61.5)	-	
Primary tumour	Duodenum	28/34	23.8 (7.5 – 40.1)	-	(0.179)
	Jejunum	5/7	28.6 (0 – 62.1)	0.50 (0.19 – 1.33)	0.164
	Ileum	4/5	60.0 (17.1 – 100)	0.45 (0.15 – 1.32)	0.146
Disease status	Locally Advanced	2/2	50.0 (0 – 100)	-	
	Metastatic	35/44	28.4 (13.9 – 42.9)	1.35 (0.32 – 574)	0.683
Chemo 1^ST^ line	Doublet	15/18	25.0 (4.2 – 45.8)	-	
	Triplet	19/23	34.1 (12.5 – 55.7)	1.05 (0.53 – 2.09)	0.884
Chemo 2^nd^ line	No	18/17	27.9 (8.1 – 47.7)	-	
	Yes	19/19	31.6 (10.6 – 52.6)	1.17 (0.61 – 2.24)	0.633
Small Bowel Obstruction	None	22/28	19.8 (2.7 – 36.9)	-	
	Present	6/8	42.9 (6.2 – 79.6)	0.69 (0.27 – 1.75)	0.437
Weight loss	None	15/23	28.6 (6.1 – 51.1)	-	
	Present	13/13	23.1 (0 – 46.0)	1.29 (0.61 – 2.73)	0.499
CEA at baseline	Normal	6/9	66.7 (35.9 – 97.5)	-	
	Abnormal	25/27	12.8 (0 – 26.3)	3.18 (1.23 – 8.19)	0.017
Hemoglobin	Normal	8/10	48.0 (15.9 – 80.1)	-	
	Abnormal	25/29	20.5 (4.6 – 36.4)	1.88 (0.83 – 4.24)	0.130
Albumin	Normal	17/22	44.4 (22.1 – 66.7)	-	
	Abnormal	16/17	6.7 (0 – 19.4)	2.29 (1.12 – 4.66)	0.023
LDH	Normal	14/18	34.4 (10.9 – 57.9)	-	
	Abnormal	15/16	20.0 (0 – 40.2)	1.48 (0.70 – 3.15)	0.310
Alk.Ph	Normal	18/23	34.8 (13.0 – 56.6)	-	
	Abnormal	15/16	18.8 (0 – 38.0)	1.32 (0.66 – 2.62)	0.437
ALT	Normal	25/30	31.2 (13.6 – 48.8)	-	
	Abnormal	7/8	16.7 (0 – 45.7)	1.95 (0.82 – 4.61)	0.131
Platelets	Normal	17/24	43.6 (21.8 – 65.4)	-	
	Abnormal	17/17	11.8 (0 – 27.1)	2.32 (1.14 – 4.73)	0.020

**Table 6:** **LS-SBA - overall survival by prognostic factors (only patient who received first line chemotherapy)**

		Number events/Number subjects	1 Year survival (95% CI)	Hazard ratio (95% CI)	p-value
Gender	Male	16/24	57.1 (35.7 – 78.5)	1.06 (0.53 – 2.11)	0.869
	Female	17/22	64.6 (43.4 – 85.8)	-	
Primary tumour	Duodenum	26/34	56.5 (38.5 – 74.5)	-	(0.130)
	Jejunum	5/7	71.4 (37.9 – 100)	0.55 (0.21 – 1.45)	0.224
	Ileum	2/5	75.0 (32.5 – 100)	0.27 (0.06 – 1.17)	0.080
Disease status	Locally Advanced	9/13	67.7 (41.4 – 94.0)	-	
	Metastatic	22/27	51.6 (31.8 – 71.4)	1.99 (0.90 - 4.42)	0.089
Chemo 1^ST^ line	Doublet	11/18	52.6 (28.5 – 76.7)	-	
	Triplet	19/23	64.8 (43.6 – 86.0)	1.67 (0.78 – 3.55)	0.185
Chemo 2^nd^ line	No	18/17	27.9 (8.1 – 47.7)	-	
	Yes	19/19	31.6 (10.6 – 52.6)	1.17 (0.61 – 2.24)	0.633
Small Bowel Obstruction	None	21/28	55.3 (35.3 – 75.3)	-	
	Present	4/8	51.4 (11.4 – 91.4)	0.55 (0.19 – 1.61)	0.274
Weight loss	None	13/23	49.4 (25.1 – 73.7)	-	
	Present	12/13	61.5 (35.0 – 88.0)	1.11 (0.50 – 2.45)	0.803
CEA at baseline	Normal	5/9	66.7 (35.9 - 97.5)	-	
	Abnormal	23/27	52.2 (32.4 – 72.0)	3.15 (1.15 – 8.60)	0.025
Hemoglobin	Normal	8/10	90.0 (71.4 – 100)	-	
	Abnormal	22/29	46.2 (26.6 – 65.8)	1.51 (0.67 – 3.43)	0.323
Albumin	Normal	15/22	80.4 (63.2 – 97.6)	-	
	Abnormal	15/17	32.1 (9.2 – 55.0)	2.15 (1.03 – 4.46)	0.041
LDH	Normal	13/18	63.6 (39.9 – 87.3)	-	
	Abnormal	13/16	53.3 (28.0 – 78.6)	1.31 (0.59 – 2.87)	0.507
Alk.Ph	Normal	16/23	70.5 (50.3 – 90.7)	-	
	Abnormal	14/16	43.8 (19.5 – 68.1)	1.45 (0.70 – 2.99)	0.320
ALT	Normal	23/30	63.4 (45.2 – 81.6)	-	
	Abnormal	6/8	31.3 (0 – 66.2)	1.96 (0.77 – 4.96)	0.156
Platelets	Normal	15/24	73.4 (55.0 – 91.8)	-	
	Abnormal	16/17	38.0 (14.1 – 61.9)	2.85 (1.34 – 6.08)	0.007

### First-line chemotherapy efficacy in LS-SBA

Of the forty six patients, who received first-line chemotherapy (median 6 cycles, range 1–12), 40 were evaluable for response assessment by RECIST criteria. ORR was found to be 50% [1(2.5%) CR, 19 (47.5%) PR]; whilst 8 (20%) and 12 (30%) were found to have SD and PD as their best response. Patents had a median follow up time of 63.7 months (95% CI = 16.5-110.9). Patients who received chemotherapy had an overall 1-year survival of 60.9% (95% CI = 45.8-76.0). When compared to patients who didn’t receive chemotherapy, the survival was significantly better (P = 0.042), as only 27.3% were alive at 1-year time point. Of the 23 patients that received triplet regimens, EOX (Epirubicin, oxaliplatin and capecitabine; n = 13), ECX (Epirubicin, cisplatin and capecitabine; n = 4), ECF (Epirubicin, cisplatin and 5-FU; n = 3), E-Carbo-F (Epirubicin, carboplatin and 5-FU; n = 2) were commonly prescribed regimens in the order of descending frequency; one patients received Mitomycin C in combination with carboplatin and 5-FU. Whilst of the 18 patients who were offered doublet chemotherapy combinations, the commonly prescribed regimens in the order of descending frequency included CAPOX (capecitabine and oxaliplatin; n = 6), FOLFOX (5-FU and oxaliplatin; n = 4), FOLFIRI (5-FU and irinotecan; n = 3), and capecitabine with Mitomycin C (n = 3); the remaining two patients received gemcitabine with capecitabine, and gemcitabine in combination with bevacizumab respectively. When compared, no statistically significant difference was found in the OS of patients treated with triplet (23/41) vs. doublet (18/41) regimens in univariate analysis (p = 0.185) (Figure 3).

### Second-line chemotherapy efficacy in LS-SBA

Patients who received second line chemotherapy were found to have a RR of 63.2%; however, no significant improvement in PFS or OS were observed when patients who received second line chemotherapy were compared to those who didn’t receive second-line chemotherapy (Tables 5 and 6).

## Discussion

SBA is a relatively rare but highly aggressive disease. There is paucity of literature and expertise in management of this disease due to lack of prospective clinical studies evaluating the management strategies in SBA. Although the disease remains rare, there has been a trend towards better and early diagnosis of SBA in recent times due to the advancements in diagnostic investigations including video-capsule endoscopy and/or double balloon enteroscopy. Our series is one of the largest to consider clinical and pathological characteristics of SBA. In our series, patients were divided into two clinically relevant groups; we sought to gain clinically meaningful information about both groups within the limitations of retrospective nature of this study.

In ES-SBA, the role of adjuvant chemotherapy remains unclear due to lack of any phase III clinical trials evaluating the role of adjuvant chemotherapy. Our data and that of others, however, show that even after curative resection, more than half of the patients with ES-SBA succumb to metastatic disease [4,8]. The role of adjuvant chemotherapy has often been debated as some physicians considered the disease similar to CRC, where the role of adjuvant chemotherapy is better defined while others treated it as upper GI cancer where although the role of peri-operative chemotherapy is well established, the role of adjuvant chemotherapy remains debatable. However, more recently some studies [9,10] have shown the molecular similarities between SBA and CRC. Therefore keeping in view the aggressive disease biology and molecular similarities with CRC, logically there might be a role for considering adjuvant chemotherapy in ES-SBA. Our limited knowledge from previously published retrospective work however shows no convincing evidence in favour of adjuvant chemotherapy [11-13]. The only positive experience from a single-centre study demonstrated improvement in disease free survival (HR 0.27; 95% CI 0.07–0.98, p *=* 0.05) but not in OS (HR 0.47; 95% CI 0.13–1.62, p = 0.23) in a multivariate analysis [14]. The present study also failed to demonstrate survival advantage in favour of adjuvant chemotherapy. This however, we think may be due to selection bias and retrospective data collection; patients with high risk disease tend to be offered chemotherapy which may impair the comparison between the patients who received or didn’t receive chemotherapy. A prospective international phase-III study (the BALLAD study), which is currently in setup, promoted by the International Rare Cancer Initiative, will be able to address the role of adjuvant chemotherapy in SBA.

Previous studies have determined pT4 tumour stage, poor histological differentiation, positive resection margins, LVI and low number of LNH as the poor prognostic factors in ES-SBA [11,15,16]. There is conflicting evidence as to whether duodenal primary site is associated with poor outcome [9,14]. In the present series, poor histological differentiation, LVI and abnormal CEA at presentation were prognostic of OS. Tumour site and LNH were not found to be prognostic of survival. RFS and OS in our series were similar or slightly better than those published previously. 5-year RFS and OS were 43.1% and 47.7% respectively in our series. These results may reflect the high volume of GI surgeries and associated expertise in our centre, along with higher median follow up time compared to some previously published studies. Furthermore, patients who were deemed resectable, most of the times underwent R0 resection of their disease; although due to small number of patients with R1 resection in our series, the comparison between the outcomes of R0 (n = 44) and R1 (n = 4) resection didn’t reach statistical significance. Of note, of the 27 patients who received adjuvant chemotherapy in our series, 3 had R1 resection; one patient with R1 resection did not receive adjuvant chemotherapy.

The role of chemotherapy in LS-SBA is relatively better defined. Several retrospective series [11,17-26] have shown the efficacy of chemotherapy in LS-SBA, with RR of 7-42%, PFS of 3.2-8.6 months and OS of 9.0-17.8 months. Notably, combination chemotherapy has been reported to be associated with better outcomes compared to single agent chemotherapy; although no head to head comparison has been made and the data should be interpreted with caution. Only a handful of phase II prospective studies have examined the role of palliative chemotherapy in LS-SBA; whilst slightly better RR were observed in those trials compared to retrospective data, PFS and OS were found to be similar to what has been observed in retrospective studies [27-29]. In the present series, our data are comparable to previously published data. It is noteworthy that in our series, most of the patients had metastatic disease (54/59) at presentation; additionally 23 patients presented with relapsed disease. This may overall represent difficult disease biology to treat. Patients with SBA have been traditionally treated with variety of chemotherapy regimens due to lack of randomised phase III data and incertitude about the molecular behaviour of SBA until recently. We therefore compared the impact of triplet (23/41) vs. doublet (18/41) chemotherapy regimens and found no statistically significant difference in the PFS or OS between these treatments in univariate analysis. Moreover, the RR to doublet (41.2%) and to the triplet (52.2%) regimens was found to be statistically insignificant (p = 0.491). The heterogeneity of the treatment regimens in this setting reflects the historical debate about the nature of SBA. To our knowledge, this is the first series reporting the comparison between the outcomes of the patients treated with triplet or doublet chemotherapy regimens. Our results either reflect the absence of true difference in the outcomes when treated with triplet or doublet therapy or may indeed simply reflect the low power of the study to demonstrate a statistically significant difference. Patients who received systemic chemotherapy in the present study had a significantly higher 1 year-survival of 60.9%, compared to 27.3% for those who didn’t receive systemic treatment (p = 0.042). These results however should be interpreted with caution due to retrospective nature of the study and inherent bias towards selection of patients who were offered chemotherapy.

The prognostic factors including poor histological differentiation and abnormal CEA at presentation have been established as poor prognostic factors from the previously published literature in LS-SBA. Our series was in agreement with these established findings; additionally we found that low albumin and abnormal platelet count were prognostic of worse outcome for both PFS and OS. Abnormal platelet count has been considered as an important prognostic factor in CRC based on the data from UK COIN study [30], whilst we have previously reported an association of abnormal albumin with poor outcomes in upper GI cancers [31].

In the current study although we didn’t aim to analyse the histopathological and molecular biomarkers, more recently, it has been suggested that SBA has striking molecular similarities with CRC [9]; this perhaps suggests that much better outcomes should be achieved in SBA if more prospective randomised studies were designed to inform treatment strategies. Acknowledging the impact of extrapolation of data from other cancers in SBA and retrospective nature of most of the published literature, we believe that there is still significant scope of improvement in outcome of patients with SBA. We acknowledge the limitations of the current study including, single centre experience, small sample size and inherent selection bias that is associated with retrospective studies. We therefore feel that prospective clinical studies incorporating molecular targeted therapies coupled with development of robust biomarkers will help achieving better outcomes in this disease.

## Conclusion

SBA is a rare but highly aggressive disease. Due to paucity of available data, and difficulties in setting up large randomised clinical trials, retrospective data could be valuable in determining the outcome from the disease and indeed providing more useful information to the treating physicians. Our dataset is one of the largest examinations of this disease in both early stage and advanced setting. Although patients treated in our series didn’t appear to have driven any meaningful benefit from adjuvant chemotherapy; patients with advanced SBA appear to derive benefit from systemic chemotherapy. Prospective clinical trials are however required to define optimal chemotherapy regimens. Clinical prognostic factors evaluated in our series may be useful for stratification and eligibility considerations in future clinical trials.

## References

[1] Bilimoria KY, Bentrem DJ, Wayne JD, Ko CY, Bennett CL, Talamonti MS (2009). Small bowel cancer in the United States: changes in epidemiology, treatment, and survival over the last 20 years. Ann Surg.

[2] Siegel R, Ma J, Zou Z, Jemal A (2014). Cancer statistics, 2014. CA Cancer J Clin.

[3] Faivre J, Trama A, De Angelis R, Elferink M, Siesling S, Audisio R (2012). Incidence, prevalence and survival of patients with rare epithelial digestive cancers diagnosed in Europe in 1995–2002. Eur J Cancer.

[4] Aparicio T, Zaanan A, Svrcek M, Laurent-Puig P, Carrere N, Manfredi S (2014). Small bowel adenocarcinoma: epidemiology, risk factors, diagnosis and treatment. Dig Liver Dis.

[5] Raghav K, Overman MJ (2013). Small bowel adenocarcinomas–existing evidence and evolving paradigms. Nat Rev Clin Oncol.

[6] Center MM, Jemal A, Ward E (2009). International trends in colorectal cancer incidence rates. Cancer Epidemiol Biomarkers Prev.

[7] Duffaud F, Therasse P (2000). New guidelines to evaluate the response to treatment in solid tumors. Bull Cancer.

[8] Overman MJ (2013). Rare but real: management of small bowel adenocarcinoma. Am Soc Clin Oncol Educ Book.

[9] Aparicio T, Svrcek M, Zaanan A, Beohou E, Laforest A, Afchain P (2013). Small bowel adenocarcinoma phenotyping, a clinicobiological prognostic study. Br J Cancer.

[10] Cunningham D, Atkin W, Lenz HJ, Lynch HT, Minsky B, Nordlinger B (2010). Colorectal cancer. Lancet.

[11] Halfdanarson TR, McWilliams RR, Donohue JH, Quevedo JF (2010). A single-institution experience with 491 cases of small bowel adenocarcinoma. Am J Surg.

[12] Agrawal S, McCarron EC, Gibbs JF, Nava HR, Wilding GE, Rajput A (2007). Surgical management and outcome in primary adenocarcinoma of the small bowel. Ann Surg Oncol.

[13] Wu TJ, Yeh CN, Chao TC, Jan YY, Chen MF (2006). Prognostic factors of primary small bowel adenocarcinoma: univariate and multivariate analysis. World J Surg.

[14] Overman MJ, Kopetz S, Lin E, Abbruzzese JL, Wolff RA (2010). Is there a role for adjuvant therapy in resected adenocarcinoma of the small intestine. Acta Oncol.

[15] Overman MJ, Hu CY, Wolff RA, Chang GJ (2010). Prognostic value of lymph node evaluation in small bowel adenocarcinoma: analysis of the surveillance, epidemiology, and end results database. Cancer.

[16] Nicholl MB, Ahuja V, Conway WC, Vu VD, Sim MS, Singh G (2010). Small bowel adenocarcinoma: understaged and undertreated?. Ann Surg Oncol.

[17] Ouriel K, Adams JT (1984). Adenocarcinoma of the small intestine. Am J Surg.

[18] Jigyasu D, Bedikian AY, Stroehlein JR (1984). Chemotherapy for primary adenocarcinoma of the small bowel. Cancer.

[19] Crawley C, Ross P, Norman A, Hill A, Cunningham D (1998). The Royal Marsden experience of a small bowel adenocarcinoma treated with protracted venous infusion 5-fluorouracil. Br J Cancer.

[20] Dabaja BS, Suki D, Pro B, Bonnen M, Ajani J (2004). Adenocarcinoma of the small bowel: presentation, prognostic factors, and outcome of 217 patients. Cancer.

[21] Locher C, Malka D, Boige V, Lebray P, Elias D, Lasser P (2005). Combination chemotherapy in advanced small bowel adenocarcinoma. Oncology.

[22] Fishman PN, Pond GR, Moore MJ, Oza A, Burkes RL, Siu LL (2006). Natural history and chemotherapy effectiveness for advanced adenocarcinoma of the small bowel: a retrospective review of 113 cases. Am J Clin Oncol.

[23] Overman MJ, Kopetz S, Wen S, Hoff PM, Fogelman D, Morris J (2008). Chemotherapy with 5-fluorouracil and a platinum compound improves outcomes in metastatic small bowel adenocarcinoma. Cancer.

[24] Zaanan A, Costes L, Gauthier M, Malka D, Locher C, Mitry E (2010). Chemotherapy of advanced small-bowel adenocarcinoma: a multicenter AGEO study. Ann Oncol.

[25] Zaanan A, Gauthier M, Malka D, Locher C, Gornet JM, Thirot-Bidault A (2011). Second-line chemotherapy with fluorouracil, leucovorin, and irinotecan (FOLFIRI regimen) in patients with advanced small bowel adenocarcinoma after failure of first-line platinum-based chemotherapy: a multicenter AGEO study. Cancer.

[26] Tsushima T, Taguri M, Honma Y, Takahashi H, Ueda S, Nishina T (2012). Multicenter retrospective study of 132 patients with unresectable small bowel adenocarcinoma treated with chemotherapy. Oncologist.

[27] Xiang XJ, Liu YW, Zhang L, Qiu F, Yu F, Zhan ZY (2012). A phase II study of modified FOLFOX as first-line chemotherapy in advanced small bowel adenocarcinoma. Anticancer Drugs.

[28] Overman MJ, Varadhachary GR, Kopetz S, Adinin R, Lin E, Morris JS (2009). Phase II study of capecitabine and oxaliplatin for advanced adenocarcinoma of the small bowel and ampulla of Vater. J Clin Oncol.

[29] Gibson MK, Holcroft CA, Kvols LK, Haller D (2005). Phase II study of 5-fluorouracil, doxorubicin, and mitomycin C for metastatic small bowel adenocarcinoma. Oncologist.

[30] Adams RA, Meade AM, Seymour MT, Wilson RH, Madi A, Fisher D (2011). Intermittent versus continuous oxaliplatin and fluoropyrimidine combination chemotherapy for first-line treatment of advanced colorectal cancer: results of the randomised phase 3 MRC COIN trial. Lancet Oncol.

[31] Khan K, Ang JE, Starling N, Sclafani F, Shah K, Judson I (2014). Phase I trials in patients with relapsed, advanced upper gastrointestinal carcinomas: experience in a specialist unit. Gastric Cancer.

